# Using Apache Spark on genome assembly for scalable overlap-graph reduction

**DOI:** 10.1186/s40246-019-0227-1

**Published:** 2019-10-22

**Authors:** Alexander J. Paul, Dylan Lawrence, Myoungkyu Song, Seung-Hwan Lim, Chongle Pan, Tae-Hyuk Ahn

**Affiliations:** 1 Bioinformatics and Computational Biology Program, Saint Louis University, St. Louis, MO USA; 2 Computational and Systems Biology Program, Washington University in St. Louis, St. Louis, MO USA; 3 Department of Computer Science, University of Nebraska at Omaha, Omaha, NE USA; 4 National Center for Computational Sciences, Oak Ridge National Laboratory, Oak Ridge, TN USA; 5 School of Computer Science, University of Oklahoma, Norman, OK USA; 6 Department of Computer Science, Saint Louis University, St. Louis, MO USA

**Keywords:** Graph reduction, Apache spark, Genome assembly, Cloud computing, Overlap-layout-consensus

## Abstract

**Background:**

De novo genome assembly is a technique that builds the genome of a specimen using overlaps of genomic fragments without additional work with reference sequence. Sequence fragments (called reads) are assembled as contigs and scaffolds by the overlaps. The quality of the de novo assembly depends on the length and continuity of the assembly. To enable faster and more accurate assembly of species, existing sequencing techniques have been proposed, for example, high-throughput next-generation sequencing and long-reads-producing third-generation sequencing. However, these techniques require a large amounts of computer memory when very huge-size overlap graphs are resolved. Also, it is challenging for parallel computation.

**Results:**

To address the limitations, we propose an innovative algorithmic approach, called **S**calable **O**verlap-graph **R**eduction **A**lgorithms (SORA). SORA is an algorithm package that performs string graph reduction algorithms by Apache Spark. The SORA’s implementations are designed to execute de novo genome assembly on either a single machine or a distributed computing platform. SORA efficiently compacts the number of edges on enormous graphing paths by adapting scalable features of graph processing libraries provided by Apache Spark, GraphX and GraphFrames.

**Conclusions:**

We shared the algorithms and the experimental results at our project website, https://github.com/BioHPC/SORA. We evaluated SORA with the human genome samples. First, it processed a nearly one billion edge graph on a distributed cloud cluster. Second, it processed mid-to-small size graphs on a single workstation within a short time frame. Overall, SORA achieved the linear-scaling simulations for the increased computing instances.

## Background

Next-generation sequencing (NGS) refers high-throughput and in-parallel DNA sequencing technologies developed around 2007 after the Sanger DNA sequencing method first emerged in 1977 [1]. NGS technologies are different from the long dominated Sanger method in that NGS provides massive sequencing analysis with being extremely high-throughput from multiple samples at much reduced cost. Following the introduction of NGS techniques [2, 3], prodigious changes have occurred in the biological and biomedical sciences, specifically in genomics [3]. With reductions in sequencing cost and increased throughput, read length, and read accuracy NGS has drastically recast DNA sequencing; however, NGS requires a significant body of sequencing data for analysis. As reported by previous studies, NGS faces several limitations [4]. For example, in comparison to the sequence length generated by first-generation Sanger sequencing (500 ∼1000bp), fragmented DNA sequences (i.e. reads) are generally shorter (50 ∼300bp). Recently developed third-generation sequencing techniques such as Pacific Bio-sciences (PacBio) and Oxford NanoPore provide much longer reads (up to 2 Mbp) to the considerable benefit of the assembly. However, NGS remains dominant due to its low cost and error rate.

Two different types are generally used for genome assembly: de novo assembly and reference-based assembly. De novo assembly is the process of finding overlaps and merging reads to complete genome sequence that is inherently challenging but essential to bioinformatics research [5]. Reference-based assembly can construct a new specimen genome with help of similar assembled genome. Third-generation sequencing can produce reads having nearly similar size of bacterial genomes that usually are few Mbp long, but cannot generate full sequences of eukaryotic genomes up to several Gbp of length. For example, the haploid human genome size is over 3 Gbp and the Genome Reference Consortium Human Build 38 patch release 13 (GRCh38.p13) is the most recently released human genome assembly [6].

The elaboration of genome assembly stems from multiple issues including heterozygosity and ploidy, affected mainly by the length and numbers of the reads. To assemble such large datasets, most de novo assembly programs are highly sensitive to the changes in time and space complexity. To account for both sensitivity and speed, most de novo genome assemblers commonly employed two assembly paradigms. One is overlap-layout-consensus (OLC) algorithm and the other is de Bruijin graph (DBG) [7]. During the first-generation Sanger sequencing technique era, OLC approaches, i.e., Celera [8], reached accuracy adequate to accommodate the low sequencing depth and longer reads output. Newbler [9] that was designed for second-generation Roche / 454 Life Sciences sequences also adapted the OLC approach. The majority of OLC-based genome assemblies produce the sequence assembly of whole, complex genomes using below steps. First, finding **O**verlaps between fragments or among all reads by using a graph model. Second, using the overlay-graph to construct a stretched **L**ayout. Third, establishing the most probable **C**onsensus sequence.

Various alternate approaches using DBG concept were proposed to assemble a genome with noticeably high-throughput and short reads from NGS technologies. Under NGS, DBG-based assemblers have been commonly employed to degrade reads into *k*-mers where a *k*-mer is a subsequence of a fixed-length, *k*. Various DBG-based assemblers including AbySS [10], Velvet [11] and SOAPdenovo [12] utilize memory-efficient DBG traversal to lessen the memory footprint of assembly including an efficient identification of redundant *k*-mers. As opposed to the less computationally efficient (e.g. costly execution time and memory consumption per assembler) OLC-based approaches, most DBG-based assemblers reduce dependency on sequencing depth using a genome-sized graph at the cost of a larger memory overhead. The DBG-based approach achieves comparably fast overlapping computation for high-throughput short reads, while the OLC-based approach performs more advantageously for longer reads. Most of the DBG-based techniques adapt hashing algorithms that have a chance to acquire higher relative error rates but usually perform faster than the OLC-based approaches [13].

Lately, probabilistic algorithms utilizing the MinHash technique have been developed to efficiently identify multiple overlaps between long, noisy reads from third-generation sequencing data [14, 15]. Canu, as a successor of the Celera assembler, was designed for long and noisy single-molecule sequences [15]. However, the computationally expensive overlap graphs produced by the assembly of raw or processed sequences must be simplified or reduced. Several MPI-based scalable assemblies were proposed previously; including Abyss [10], Ray [16], and SWAP2-Assembler [17]. Apache Spark serves an a general purpose and open source and distribution computing engine for cluster based computation with pre-build libraries such as GraphX, MLlib (Machine Learning library), Spark Steaming, and so on [18, 19]. Utilizing data intensive cluster computation, Apache Spark processes large scale data quickly though efficient in-memory computation. Unlike the Apache Hadoop, a conventional cloud-based distributed processing framework, Spark can accelerate computational performance by up to 100 times compared to the Hadoop especially for interactive jobs and iterative analytics by cacheing datasets in memory. MPI is a popular framework for high performance parallel computing, but Spark provides an in-memory implementation of MapReduce that is widely used in the big data industry.

Due to the extensive memory and processing time required, the analysis of reads with significant overlap is not easily parallelized. To address these challenges, we propose a novel OLC-based algorithmic approaches for genome assembly, called **S**calable **O**verlap-graph **R**eduction **A**lgorithms (SORA) by leveraging Apache Spark especially with the GraphX and GraphFrames libraries. Using the computing engine of Apache Spark, SORA accelerates the graph reductions for genome assemblies by compacting repetitive information of sequence overlaps either in the cloud, by a local cluster system, or using a stand-alone workstation. SORA was developed as an open-source framework to provide pre-built modules for graph reduction with useful scripts for genome assembly including sequence overlap finding using BBtools (https://sourceforge.net/projects/bbmap/). SORA executes genome assembly through the use of three overlap-graph reduction algorithms: *Transitive Edge Reduction*, *Dead-End Removal*, and *Composite Edge Contraction*. It presents a short turnaround time when processing a large-scale dataset consisting of a graph with nearly one billion edges on a distributed cloud computing cluster or when processing a smaller 8 million edge graph dataset on a local computing cluster. Spaler [20] is another GraphX and Apache Spark based de novo genome assembler utilizing DBG contraction and construction, but SORA is, to our knowledge, the first proposed Spark-based scalable assembler utilizing the OLC approach. Our previous studies [21, 22] were extensively extended in this paper. In detail, two primary goals are demonstrated in our benchmark results; (1) SORA actualizes a cloud scalable de novo genome assembler through leveraging Apache Spark graph processing libraries; (2) SORAdemonstrates the applicability of cloud computing infrastructure employing graphing algorithms to genome assembly and alternative biological applications. The increasing popularity of Spark among computational researchers has also influenced our decision to use Spark [23].

The remainder of the article is organized as follows. “Methods” section describes the OLC algorithm and Apache Spark, then presents SORA’s algorithms and the implementation in detail. “Results” section describes various experiments conducted to evaluate the scalability and usability of SORA using large and small scale datasets on cloud followed by Discussion and Conclusions.

## Methods

### Overlap-Layout-Consensus

The Overlap process, the initial step of OLC, focuses on finding overlaps of all reads using all-to-all pairwise alignments. To efficiently find overlaps between reads, the prefix/suffix technique is commonly used for overlap-based genome assembly [24]. This hash table approach allows a nearly constant time search when reads are small of all reads by their prefixes and suffixes. To efficiently search all overlapping reads with a read *r*, each proper substring of minimum overlap in read *r* is found in the hash table, and every retrieved read is compared to the read *r*. Therefore, an overlap-graph that places reads as nodes and assigns edges between nodes whose corresponding reads overlap exceeds a specified cutoff is constructed by the Overlap step. As a result, the number of nodes will be proportionate to the number of unique reads, while the number of overlaps between reads will determine the number of edges.

During the Layout and Consensus steps, the manufactured overlap-graph is stretched and reduced into the most probable contiguous sequences, labeled, contigs. The Layout step acts as a Hamiltonian path problem where each read in the graph must be visited to generate longer sequences. This is a computationally challenging problem caused by a large number of unnecessary edges that are mostly produced by repeats or sequencing errors. As the final step, Consensus considers the alignment of all original reads onto the draft contigs from the Layout step and employs a straightforward majority-based consensus to improve the draft sequences. To limit extraneous edges in the graph, SORA utilizes three overlaps-graph reduction algorithms: Transitive Edge Reduction (TER), Composite Edge Contraction (CEC), and Dead-End Removal (DER) [25].

### Apache Spark

Apache Spark is a cluster-based engine that processes very large-scale datasets. As opposed to Hadoop’s on-disk data processing, Spark’s incorporated batching system handles input data streams in-memory, separates the data into batches for each node in a cluster, and produces the final stream of results in batches. For fast and scalable distributed graph-parallel computation, Apache Spark provides GraphX library that contributes a set of fundamental operations and graph abstraction models in parallel. This permits SORA to manipulate and execute queries on graphs represented as database entries. The implementation and design in SORA leverages an assortment of computational operations in GraphX for construction, graph reading, transformation, and computation. GraphX extends Spark’s Resilient Distributed Dataset (RDD) that embodies a read-only collection of objects that are partitioned over machines. If any partition of an RDD is lost, Spark rebuilds it by applying the filter on the corresponding block of the file in the file system. An RDD can be cached in memory across machines and reused in multiple MapReduce-like parallel operations.

To accommodate abstraction for manipulating structured data (e.g., tables or two-dimensional arrays), SORA uses a graph processing library called GraphFrames that is built on Spark’s DataFrame implementation to process real-time exploration of large-volume datasets. SORA leverages GraphFrames to execute pattern matching and relational queries in tandem with GraphX to speed up the most common join in iterative graph processing tasks. SORA was implemented in Scala, but the portable design of the core components allows for adaptive use with other programming languages like Java or Python with lower development costs.

### Overlap-Graph Reduction Algorithms

**Fig. 1:**
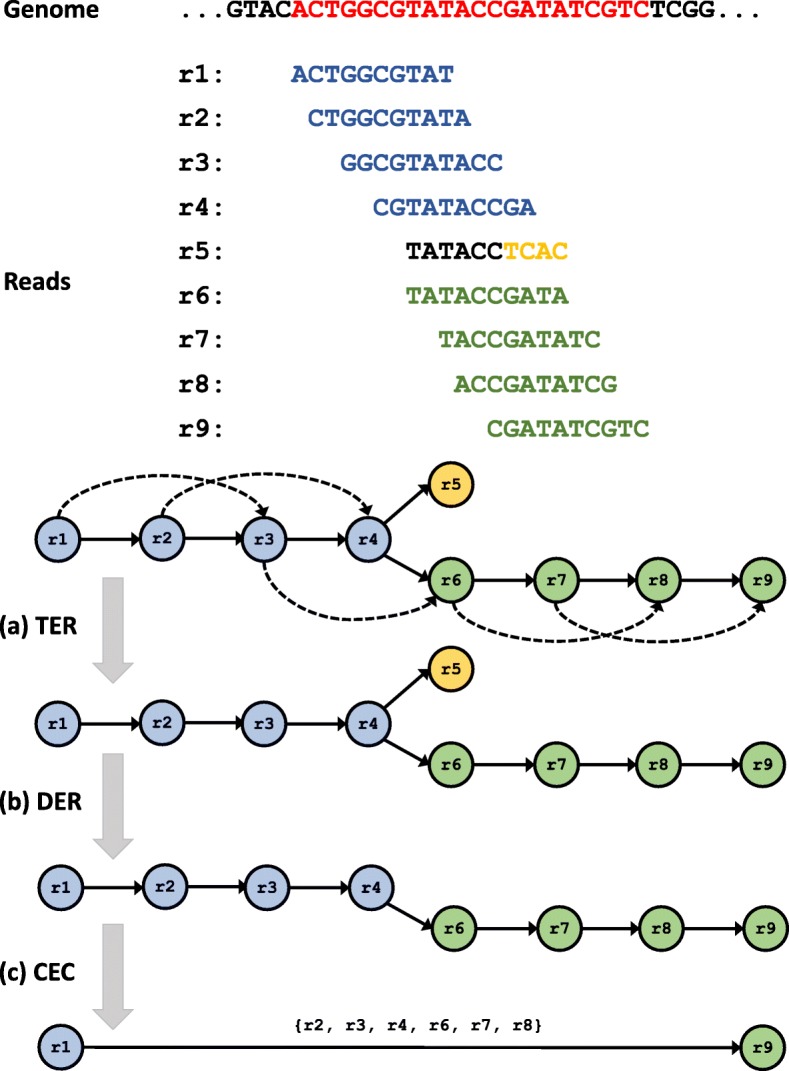
The overlap-graph reduction algorithms. **a** Transitive Edge Reduction (TER), **b** Dead-End Removal (DER), and **c** Composite Edge Contraction (CEC)

This section illustrates SORA’s adaptation of three overlap-graph reduction algorithms to the distributed cloud computing cluster utilizing Spark. Figure 1 represents the synopsis of each workflow as to how each algorithm computes overlap-graph reduction.

#### Transitive Edge Reduction

Transitive edge reduction is a method of reducing complexity in graphs and helps provide clearer contigs by eliminating extraneous paths in the graph. After finding overlaps, the initial overlap graph contains many unnecessary edges. For example, say read *a* overlaps with read *b*, which overlaps with read *c* subsequently, which results in a shorter overlap length between read *a* and read *c*. Then, the string graph edge *a*→*c* is unnecessary because one can use the edges *a*→*b*→*c* without *a*→*c* to obtain the same sequence. The edge *a*→*c* is then identified as a transitive edge and is deleted. Removing all transitive edges significantly simplifies the overlap graph without losing information for genome assembly.


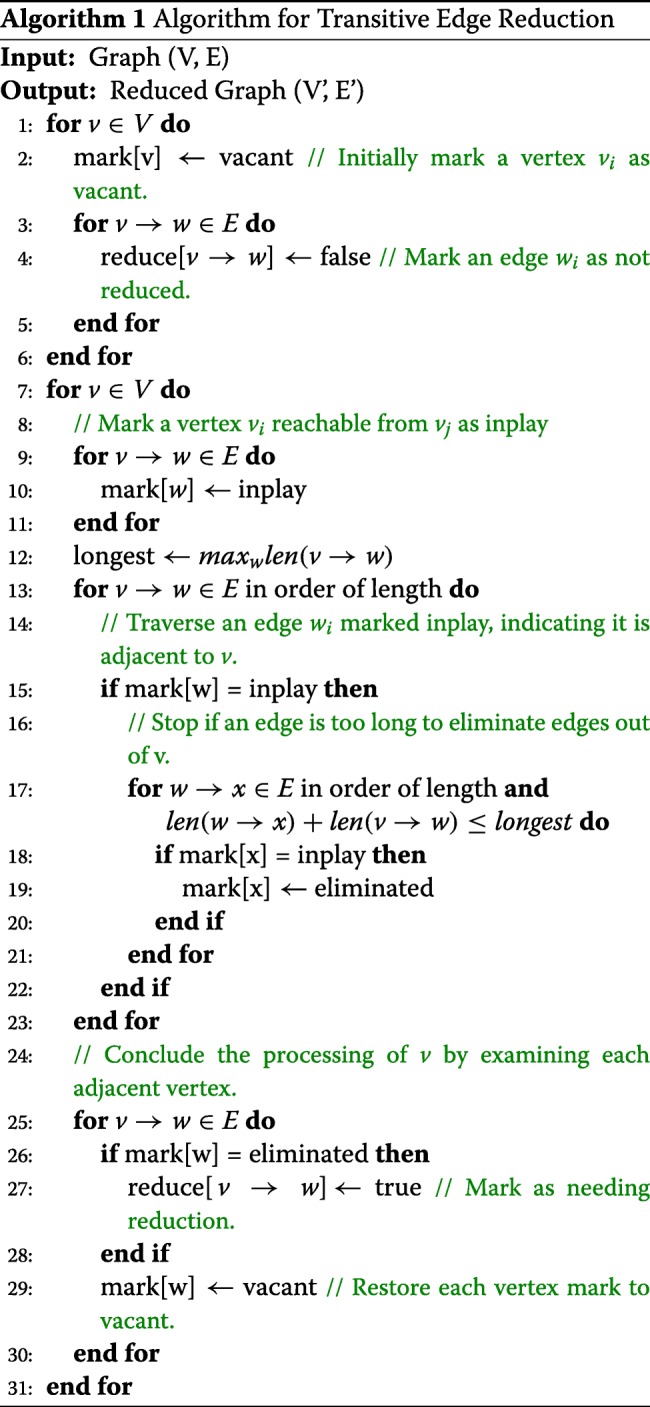



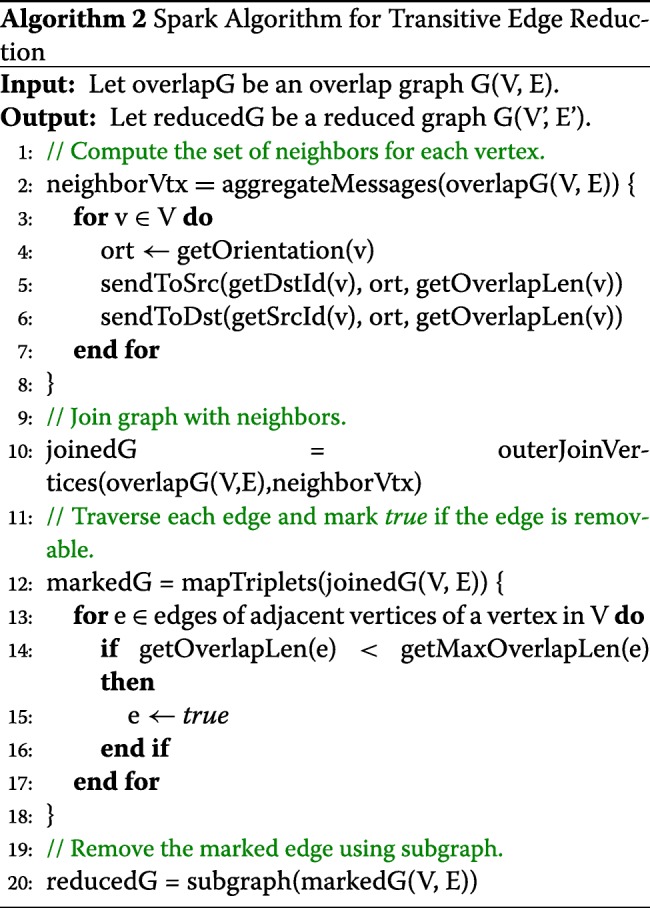


The general transitive edge reduction algorithm takes *O*(*ED*) time where *E* is the number of edges and *D* is the maximum out degree for the read, but Myer proposed a linear *O*(*E*) expected time transitive reduction algorithm shown in Algorithm 1 [25]. After the initial marking of every vertex and all related edges in the graph, each vertex is then investigated to find eliminable edges of the vertex using the marking strategies.

In Algorithm 2 we use the GraphX library operators to implement the transitive edge reduction algorithm based on the graph-parallel abstraction. The GraphX library supports the graph-parallel computation APIs aggregateMessages(), outerJoinVertices(), mapTriplets(), subgraph(), sendToSrc(), and sendToDst(). After constructing the initial property graph from the edge properties, the aggregateMessages operator can compute the set of neighbors for each vertex and retrieve the edge properties including overlap length at the same time. The required set of neighbors can be joined with the graph using outerJoinVertices. After comparing overlap lengths of the edges for each vertex in parallel, the edges are marked as TRUE if the edges can be removed. The subgraph operator returns a new graph containing only the edges not marked for removal.

#### Dead-End Removal

Dead-End Removal (DER) eliminates short dead-ends or spurs from the graph, reduces erroneous reads, and decreases the graph complexity. The short dead-end paths are mostly caused by sequencing errors and false-positive joins of overlapping of chimeric sequences. Most assemblers identify the dead-ends by considering short length edges with low-depth coverage to be dead-ends. The DER algorithm iterates over all reads, then stamps the edges if the reads have only one incoming edge and the edges are short with low coverage.


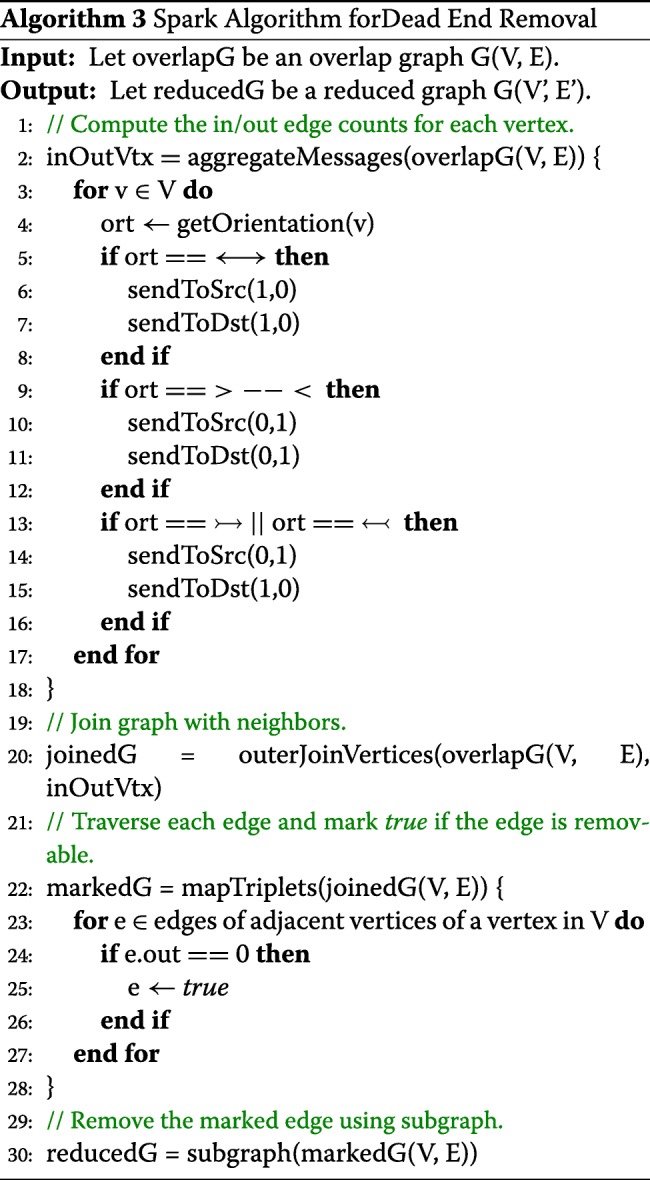


Algorithm 3 describes the DER algorithm based on the GraphX operators. Algorithm 3 takes as input the reduced graph that Algorithm 2 has produced as the output and executes the aggregateMessages operator to compute the number of edges going in and out of each vertex depending on the orientation of the edge. This information can be joined with the input reduced graph by using outerJoinVertices. In parallel, if the number of outgoing edges from a node is zero and the edge can be removed mark the edge TRUE. The subgraph operator returns a new graph with the edges marked TRUE removed.


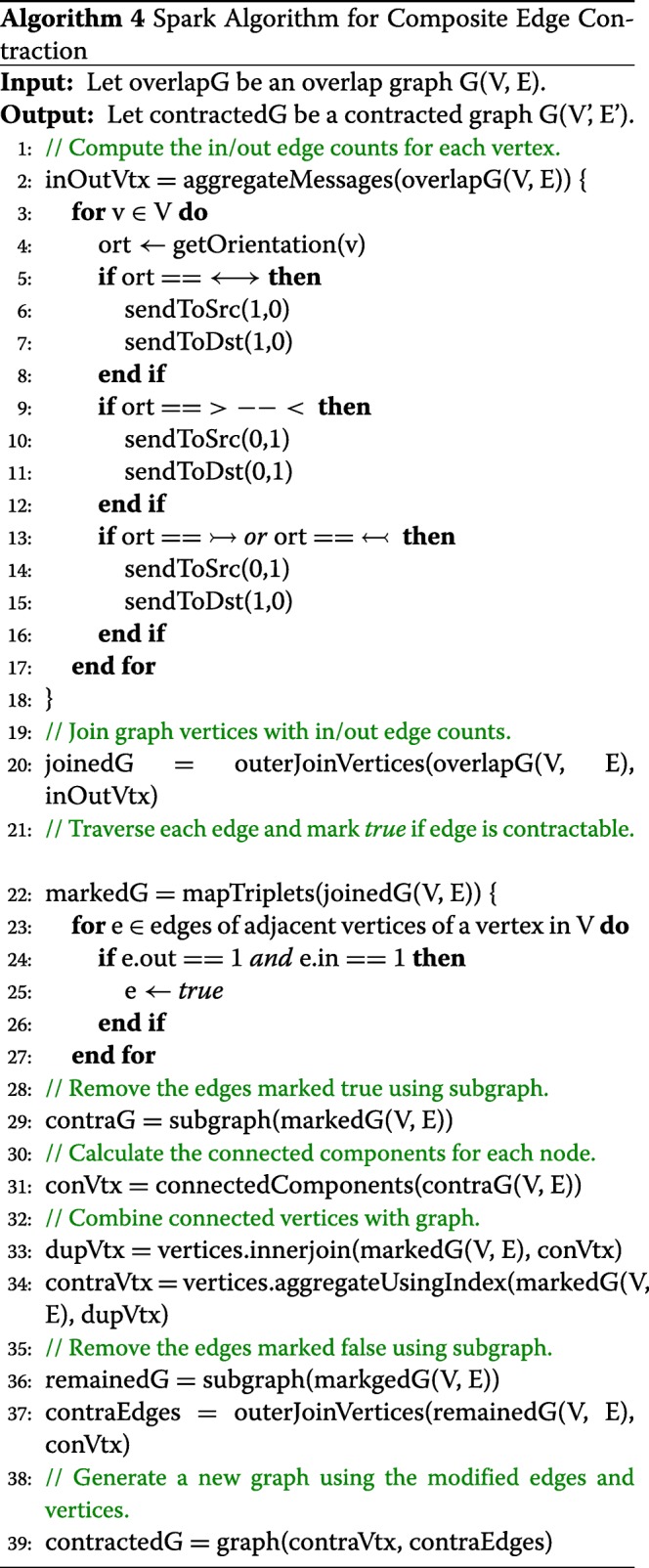


#### Composite Edge Contraction

Composite Edge Contraction (CEC) reduces the computational complexity by processing larger volumes of data in the graph. Especially, CEC merges vertices guaranteed to process the graph without loss of information. In the case of Overlap-layout-consensus (OLC), a read is represented for branching to two additional reads which deviate from each other at least one nucleotide, both of which then overlap back to the same read. In contrast to OLC, the CEC algorithm simplifies the path analysis by removing redundancy and reducing complexity of the graph, considering only the contractible edges without loss of important information for the genome assembly. To simplify the overlap graph, a simple vertex, *r*, along with its in-arrow edge (*u*, *r*) and out-arrow edge (*r*,*w*), are replaced by a composite edge (*u*,*w*) in the overlap graph.

Algorithm 4 describes the composite edge contraction by using the operators of the graph-parallel computations provided by GraphX and GraphFrames. After receiving the reduced graph from Algorithm 3, the operator aggregateMessages computes the number of edges going in and out of each vertex depending on the orientation of the edge. The result of a processed set of vertices and edges is integrated with the input reduced graph by using the operator outerJoinVertices. The operator mapTriplets is parallelized to investigate the edges of each adjacent vertex to determine whether the vertex only includes a pair of incoming and outgoing edges. It then marks the edge TRUE if they can be contracted. The subgraph operator returns a new graph with only the contractable edges.

The operator connectedComponent identifies the connection relationship among contractible vertices and produces the vertex information with the vertex IDs for the connected contractible subgraphs. Given the contractible vertex information, the operator innerJoin performs an inner join between each contractible and internal vertex to produce a set of the new vertex properties, which is used in the operator aggregateUsingIndex to aggregate the contracted vertices ensuring consistency by joining the IDs among vertices. Then, the operator subgraph filters out the edges marked FALSE to remove the contractible edges from the original graph. Based on the refined vertex set, the operator outerJoinVertices generates the contracted edges, which parameterize the operator graph to construct a new reduced graph.

## Results

**Fig. 2:**
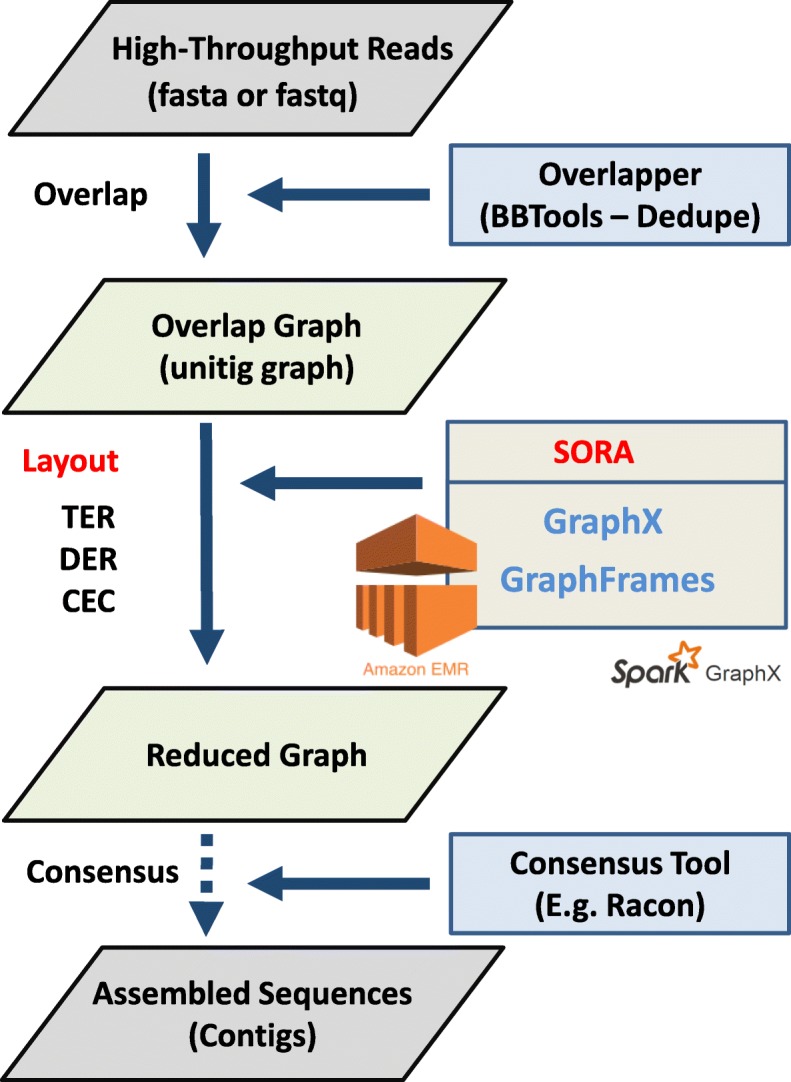
Overview of analysis pipeline using SORA. SORA pipeline for genome assembly

Figure 2 shows a practical pipeline of genome assembly using SORA. In our experiments, we applied three overlap-graph reduction algorithms (Transitive Edge Reduction, Dead-End Removal, and Composite Edge Contraction) in SORA to three different types of benchmark datasets.

### Three Data Sets

For the first experiment described in Section 6, we downloaded a metagenomics dataset from the Sequence Read Archive at the National Center for Biotechnology Information (NCBI) [24]. The accession number is SRX200676. The metagenomics dataset is considerably large containing mixed DNA from 64 diverse bacterial and archaeal microorganisms. The combined DNA was sequenced using Illumina HiSeq [26]. For the second experiment described in Section 6, we obtained a single genome dataset of *Conyza canadensis* (also known as horseweed) processed by the Illumina HiSeq sequencing system [27]. For the third experiment described in Section 6, we downloaded a human genome dataset provided by the 1000 Genome Project data portal (ISGR: The International Genome Sample Resource http://www.internationalgenome.org/). Sample ID is NA12878 (http://www.internationalgenome.org/data-portal/sample/NA12878) and we downloaded 3 files of whole genome sequencing (WGS) from the European Bioinformatics Institute (EBI) (ftp://ftp.sra.ebi.ac.uk/vol1/fastq/SRR622/SRR622461/-SRR622461_1.fastq.gz,ftp: //ftp.sra.ebi.ac.uk/vol1/fastq/SRR622/SRR622461/-SRR622461_2.fastq.gz,ftp://ftp.sra.ebi.ac.uk/vol1/fastq/SRR622/SRR622461/SRR622461.fastq.gz).

### Metagenomics Dataset Analysis

We evaluated the scalability of SORA by applying the overlap-graph reduction algorithms to the metagenomics dataset that is extremely large to check the performance capability of SORA. In the experiment, we observed that SORA significantly reduced the number of reads in the metagenomics datasets, which consequently allows binning of the contigs to reconstruct genomic bins more quickly and efficiently. The benchmark has been performed on Amazon Web Service (AWS) Elastic Computing Cloud (EC2) with 15 virtual instances whether each instance (m4.xlarge) has 2.3 GHz Intel Xeon E5-2686 v4 (Broadwell) processors (4 vCPU) and 16 GB memory.

#### Overlap Graph Construction

The sequence dataset obtained from NCBI contains 109 million paired-end reads roughly and 0.4 million single-end reads with 100-bp read length. Sequence reads that are shorter than 60bp and containing multiple N character were removed using Sickle (https://github.com/najoshi/sickle). BBNorm (https://sourceforge.net/projects/bbmap) was used for error correction with the default settings. These are the same techniques used for the OMEGA analysis [24].

#### Transitive Edge Reduction

In the experiment with the metagenomics dataset, Transitive Edge Reduction (TER) algorithm performed a drastic reduction on the number of edges in the graph. In Table 1, the reduction results of the TER algorithm were shown using three types of data size as quarter, half, and full data sets. Given the quarter dataset that contains over 217 million edges, the TER algorithm produced the reduced graph comprising 12.5 million edges with 94.24% reduction; given the full size dataset that initially contains 868 million edges, the TER algorithm made the reduced graph comprising of 57.4 million edges with 93.39% reduction.

**Table 1:** The overlap-graph reduction results with the metagenomics dataset

Algorithm	Size	#EDGE (before)	#EDGE (after)	TIME
TER	Quarter	217,002,504	12,482,946	0.57
	Half	434,005,009	23,818,401	0.80
	Full	868,010,019	57,363,515	1.37
DER-CEC	Quarter	12,482,946	469,130	0.13
	Half	23,818,401	763,474	0.23
	Full	57,363,515	2,341,610	0.40

#EDGE denotes the number of edges of the graph and TIME the running time (hours) for the computation

**Fig. 3:**
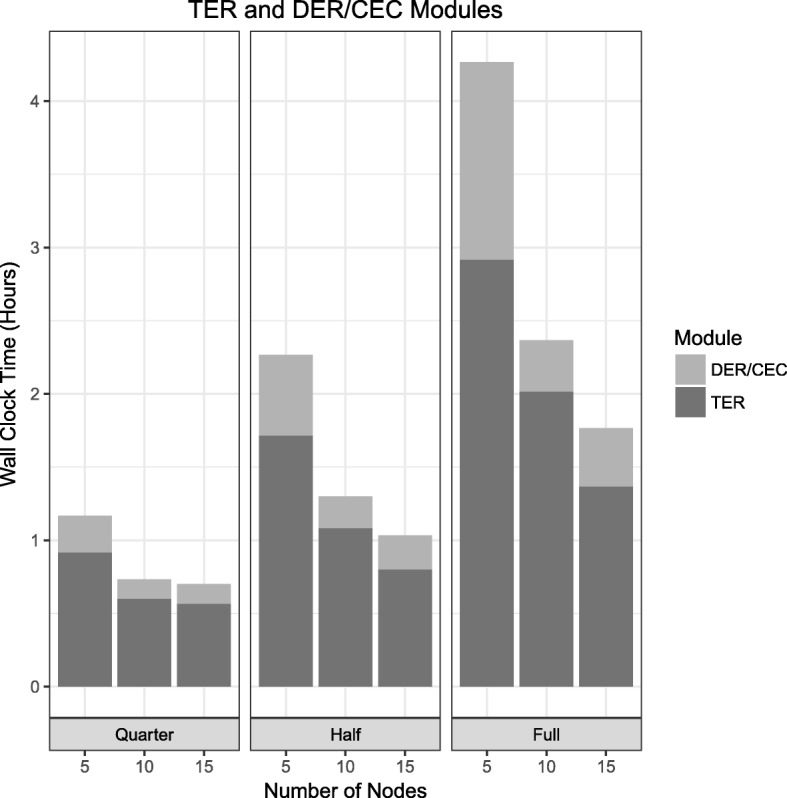
Wall-clock time comparison. Wall-clock time for different number of nodes with the different size of metagenomics datasets

Figure 3 shows the powerful scalability of the TER algorithm where the computational time decreased as the number of cluster nodes increased. For example, the TER algorithm completed the reduction of the graph module in 2.92 h using 5 cluster nodes, while completed the same task in 1.37 h with 15 cluster nodes.

#### Dead-End Removal and Composite Edge Contraction

The evaluation results of the two algorithms, Dead-End Removal (DER) and Composite Edge Contraction (CEC), using the quarter, half, and full size datasets were shown in Table 1. Given the quarter dataset that contains 12.5 million edges, the combined DER-CEC modules created the reduced graph with 0.5 million edges with 96% reduction. In addition, given the full dataset that contains 57.3 million edges, the combined DER-CEC modules resulted in the reduced graph comprising 2.3 million edges with 95.97% reduction.

Figure 3 represents the capable scalability of the combined DER-CEC algorithms by measuring each running time per different numbers of cluster nodes within the same sized dataset. In the full dataset experiment, we directly compared the running time between 5 and 15 cluster nodes. The DER-CEC algorithm completed the reduction of the graph using 5 virtual instances in 1.35 h, while fast and scalable completing in 0.4 h with 15 virtual instances.

#### Benchmark to Omega

**Fig. 4:**
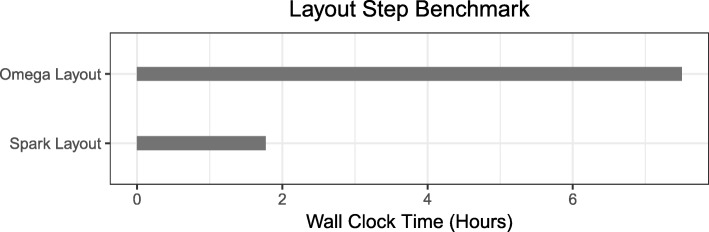
Benchmark to Omega. Shows how the analysis of the metagenomics dataset compares with Omega

To demonstrate the power of SORA’s distributed cloud computation, we benchmarked two algorithms: Omega and SORA. Omega is an string overlap-graph based metagenome assembler tool implemented in C++ [24]. We could choose another baseline application such as Spaler [20], which is a Spark-based de novo genome assembler using DBG approach, but Spaler is not publicly available for benchmarking. In Fig. 4, it shows that SORA’s computation time is only 1.77 h running time compared to Omega with 7.5 h running time. In addition to efficient speedy performance, SORA uses less amount of system memory compared to Omega since it breaks down the graph computation tasks to process them in parallel, thereby allowing more of the graph to be in memory and speeding up the analysis.

### Horseweed Dataset Analysis

To show the flexibility and usability of SORA, we applied SORA to a single genome dataset to generate a reduced graph. Total size of 72 FASTQ paired-end files is 108 GB. We used a local computational workstation that has 32 cores (Intel Xeon Processor E5-2640 V3 2.6GHz) and 128 GB of memory (DDR4 2133MHz ECC).

#### Overlap Graph Construction

**Fig. 5:**
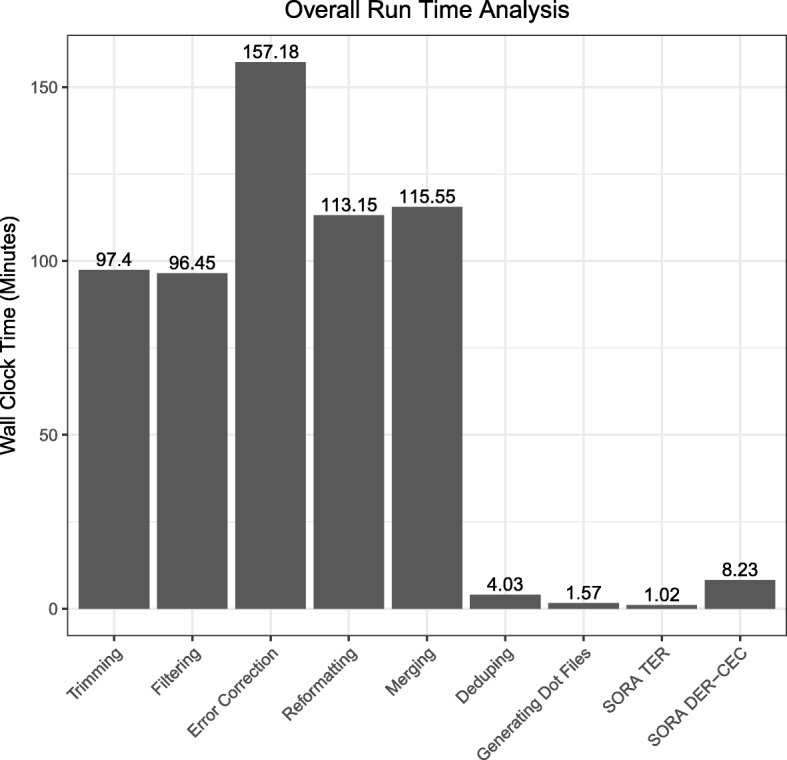
Shows the overall timing of each step from raw reads to reduced graph using SORA

To demonstrate the power of SORA for genome assembly with multiple raw reads dataset from a single genome, we implemented and incorporated multiple shell scripts into SORA to perform error correction on the genome dataset, find overlaps of the corrected reads, and generate a large overlap graph as a batch process, and thereafter executes SORA. The dataset that we tested was processed with normalization and graph construction containing 8.3 million edges. Figure 5 represents that the pipeline script including SORA completed the assembly in 9.75 h where SORA core modules (TER, DER, and CEC) only took less than 10 min.

#### Transitive Edge Reduction

Table 2 shows the assessment results using the TER algorithm with the single genome dataset that contains 8.3 million edges. After the TER algorithm, SORA produced the reduced graph that contains 5.4 million edges, which was lower reduction rate than the experiment using the metagenomic dataset since the single genome dataset is constructed less redundancies and receives fewer transitive edges potentially to be removed. Figure 5 shows that the TER algorithm completed with the best speedy performance (1.02 min execution time) with efficient memory consumption that is not requiring above 22% of overall memory usage from 128 GB total system memory.

**Table 2:** The SORA results with the horseweed dataset

	#EDGE (before)	#EDGE (after)	TIME (mins)
TER	8,259,543	5,386,287	1.02
DER-CEC	5,386,287	1,027,959	8.23

#EDGE denotes the number of edges of the graph and TIME denotes the running (wall-clock) time of the computation

#### Dead-End Removal and Composite Edge Contraction

Table 2 also shows the outcomes of overlap-graph reduction from the DER-CEC algorithms with the dataset where the graph contains 5.4 million edges generated from the TER algorithm. As we executed the algorithms DER and CEC subsequently, the DER algorithm produced the reduced graph with 4.2 million edges, whose output was fed into the CEC algorithms that completed the final graph leading to the reduced 1 million-edge graph. During this overlap-graph reduction, the DER-CEC algorithm completed the computation in 8.23 min with the maximum 37% consumption of the 128 GB total memory.

### Human Genome Dataset Analysis

In this experiment, we applied SORA to a human genome dataset to generate a reduced graph. Total size of 3 FASTQ paired-end files for one sample is 40 GB. We used a local computational workstation that has 32 cores (Intel Xeon Processor E5-2640 V3 2.6GHz) and 128 GB of memory (DDR4 2133MHz ECC) to show the ability of the SORA for a human genome sample.

#### Overlap Graph Construction

We also used a script in SORA to run BBtools trimming, filtering, error correction, merge, reformatting, merging, and finding overlaps. The duration time was approximately 1 h using 32 cores of the machine. Table 3 shows the number of edges of the overlap graph from the human genome dataset.

**Table 3:** The SORA results with the with the human genome dataset

	#EDGE (before)	#EDGE (after)	TIME (mins)
TER	18,942	10,017	1
DER-CEC	10,017	4648	2

#EDGE denotes the number of edges of the graph and TIME denotes the running (wall-clock) time of the computation

#### TER, DER, and CEC

Table 3 also shows the results of overlap-graph reduction of the TER and combined DER-CEC algorithms with the human genome dataset. The number of edges decreased to 24% of the original overlap graph. During this overlap-graph reduction, the TER, DER-CEC algorithms completed the computation in 3 min with the maximum 50% consumption of the 128 GB total memory.

## Discussion

The sequencing price continues to drop with increasing of emergence and fine tuning of novel sequencing technologies that increase the amount of sequencing data exponentially. Conventional algorithms can utilize the large influx of raw reads, but most of those algorithms require a large and expensive computing system with a large amount of computer memory. That requirement only limit to the few big labs that can afford to purchase and maintain such a powerful computing machine. SORA helps bridge this gap to small-size research labs by providing an efficient method for generating reduced graphs using distributed computing in the cloud. SORA also provides the ability to analyze any size of input data to generate novel sequenced contigs in fast turn-around time using any size of system resources.

In reference free de novo assembly, overlap-layout-consensus approach is a well-used method in low-throughput long-reads Sanger sequencing era, but can raise a problem for massive amounts of short reads that can lead many false overlaps. Therefore, it can increase the computational time and memory usage requiring for storing and analyzing large-scale graphs spawned from the massive short reads. SORA has been designed to work efficiently with these problems by using the Apache Spark engine to manage the distributed computation in the cloud or local cluster. SORA with Apache Spark efficiently uses in memory storage across multiple instances to provide a better performance compared to traditional genome assemblers.

## Conclusions

As seen in the experimental results the nearly linear scalability of SORA allows altering of the number of computational nodes as the overlap graph data size changes. By using the intrinsic attributes of each node (alignment of reads) the redundant edges in the graph can be removed using the Transitive Edge Reduction algorithm. The long stretches of multiple single edges mapped head to tail can be reduced to a single edge using the Composite Edge Contraction. Overall these algorithms provide a reduced overlap graph which allows for better contigs to be generated for de novo genome assembly.

## Abbreviations

AWS: Amazon web service
CEC: Composite edge contraction
DBG: de Bruijin graph
DER: Dead-end removal
EBI: The European Bioinformatics Institute
EC2: Elastic computing cloud
MLlib: Machine learning library
NCBI: National center for biotechnology information
NGS: Next-generation sequencing
OLC: Overlap-layout-consensus
PacBio: Pacific bio-sciences
RDD: Resilient distributed dataset
TER: Transitive edge reduction
WGS: Whole genome sequencing

## References

[1] Ansorge WJ (2009). Next-generation dna sequencing techniques. New Biotechnol.

[2] Hert DG, Fredlake CP, Barron AE (2008). Advantages and limitations of next-generation sequencing technologies: a comparison of electrophoresis and non-electrophoresis methods. Electrophoresis.

[3] Metzker Michael L. (2009). Sequencing technologies — the next generation. Nature Reviews Genetics.

[4] Wall PK, Leebens-Mack J, Chanderbali AS, Barakat A, Wolcott E, Liang H, Landherr L, Tomsho LP, Hu Y, Carlson JE (2009). Comparison of next generation sequencing technologies for transcriptome characterization. BMC Genom.

[5] Flicek Paul, Birney Ewan (2009). Sense from sequence reads: methods for alignment and assembly. Nature Methods.

[6] Schneider Valerie A., Graves-Lindsay Tina, Howe Kerstin, Bouk Nathan, Chen Hsiu-Chuan, Kitts Paul A., Murphy Terence D., Pruitt Kim D., Thibaud-Nissen Françoise, Albracht Derek, Fulton Robert S., Kremitzki Milinn, Magrini Vincent, Markovic Chris, McGrath Sean, Steinberg Karyn Meltz, Auger Kate, Chow William, Collins Joanna, Harden Glenn, Hubbard Timothy, Pelan Sarah, Simpson Jared T., Threadgold Glen, Torrance James, Wood Jonathan M., Clarke Laura, Koren Sergey, Boitano Matthew, Peluso Paul, Li Heng, Chin Chen-Shan, Phillippy Adam M., Durbin Richard, Wilson Richard K., Flicek Paul, Eichler Evan E., Church Deanna M. (2017). Evaluation of GRCh38 and de novo haploid genome assemblies demonstrates the enduring quality of the reference assembly. Genome Research.

[7] Miller Jason R., Koren Sergey, Sutton Granger (2010). Assembly algorithms for next-generation sequencing data. Genomics.

[8] Myers E. W. (2000). A Whole-Genome Assembly of Drosophila. Science.

[9] Margulies M, et al.Genome sequencing in microfabricated high-density picolitre reactors. Nature; 437(7057):376–80. 10.1038/nature03959 PMC1464427 16056220

[10] Simpson J. T., Wong K., Jackman S. D., Schein J. E., Jones S. J.M., Birol I. (2009). ABySS: A parallel assembler for short read sequence data. Genome Research.

[11] Zerbino D. R., Birney E. (2008). Velvet: Algorithms for de novo short read assembly using de Bruijn graphs. Genome Research.

[12] Li R., Zhu H., Ruan J., Qian W., Fang X., Shi Z., Li Y., Li S., Shan G., Kristiansen K., Li S., Yang H., Wang J., Wang J. (2009). De novo assembly of human genomes with massively parallel short read sequencing. Genome Research.

[13] Pop M. (2009). Genome assembly reborn: recent computational challenges. Briefings in Bioinformatics.

[14] Berlin K, Koren S, Chin CS, Drake JP, Landolin JM, Phillippy AM. Assembling large genomes with single-molecule sequencing and locality-sensitive hashing. Nat Biotechnol; 33(6):623–30. 10.1038/nbt.3238 26006009

[15] Koren S, Walenz BP, Berlin K, Miller JR, Bergman NH, Phillippy AM. Canu: scalable and accurate long-read assembly via adaptive k-mer weighting and repeat separation. Genome Res; 27(5):722–36. 10.1101/gr.215087.116 PMC5411767 28298431

[16] Boisvert S, Laviolette F, Corbeil J. Ray: Simultaneous assembly of reads from a mix of high-throughput sequencing technologies. J Comput Biol; 17(11):1519–33. 10.1089/cmb.2009.0238 PMC3119603 20958248

[17] Meng J, Seo S, Balaji P, Wei Y, Wang B, Feng S. Swap-assembler 2: Optimization of de novo genome assembler at extreme scale, 2016 45th International Conference on Parallel Processing (ICPP), Philadelphia.2016. p. 195–204. 10.1109/ICPP.2016.29.

[18] Zaharia M, Chowdhury M, Franklin MJ, Shenker S, Stoica I (2010). Spark: cluster computing with working sets. Proceedings of the 2nd USENIX conference on Hot topics in cloud computing (HotCloud’10).

[19] Zaharia M, Chowdhury M, Das T, Dave A, Ma J, McCauley M, Franklin MJ, Shenker S, Stoica I (2012). Resilient distributed datasets: a fault-tolerant abstraction for in-memory cluster computing. Proceedings of the 9th USENIX conference on Networked Systems Design and Implementation (NSDI’12).

[20] Abu-Doleh A, Catalyurek UV. Spaler: Spark and graphx based de novo genome assembler, 2015 IEEE International Conference on Big Data (Big Data), Santa Clara.2015. p. 1013–8. 10.1109/BigData.2015.7363853.

[21] Paul AJ, Lawrence D, Ahn T-H (2017). Overlap graph reduction for genome assembly using apache spark. Proceedings of the 8th ACM International Conference on Bioinformatics, Computational Biology,and Health Informatics, ACM-BCB ’17.

[22] Paul AJ, Lawrence D, Song M, Lim S, Pan C, Ahn T. Sora: Scalable overlap-graph reduction algorithms for genome assembly using apache spark in the cloud. In: 2018 IEEE International Conference on Bioinformatics and Biomedicine (BIBM): 2018. p. 718–23. 10.1109/BIBM.2018.8621546.

[23] Meng X, Bradley J, Yavuz B, Sparks E, Venkataraman S, Liu D, Freeman J, Tsai D, Amde M, Owen S, et al.Mllib: Machine learning in apache spark. J Mach Learn Res. 2016; 17(1):1235–41.

[24] Haider Bahlul, Ahn Tae-Hyuk, Bushnell Brian, Chai Juanjuan, Copeland Alex, Pan Chongle (2014). Omega: an Overlap-graph de novo Assembler for Metagenomics. Bioinformatics.

[25] Myers EW (2005). The fragment assembly string graph. Bioinformatics.

[26] Shakya Migun, Quince Christopher, Campbell James H., Yang Zamin K., Schadt Christopher W., Podar Mircea (2013). Comparative metagenomic and rRNA microbial diversity characterization using archaeal and bacterial synthetic communities. Environmental Microbiology.

[27] Peng Y, Lai Z, Lane T, Nageswara-Rao M, Okada M, Jasieniuk M, O’Geen H, Kim RW, Sammons RD, Rieseberg LH, Stewart CN (2014). De novo genome assembly of the economically important weed horseweed using integrated data from multiple sequencing platforms. Plant Physiol.

